# Retraction Note: Is early intervention using Mansoura-VV uterine compression sutures an effective procedure in the management of primary atonic postpartum hemorrhage? : a prospective study

**DOI:** 10.1186/s12884-023-05410-1

**Published:** 2023-02-04

**Authors:** Abd Elaziz A. El Refaeey, Hosam Abdelfattah, Alaa Mosbah, Anas M. Gamal, Emad Fayla, Waleed Refaie, Abdelhady Zaied, Rafik I. Barakat, Amal K. Seleem, Mohammed Maher

**Affiliations:** 1 Department of Obstetrics and Gynecology, Mansoura University, Mansoura, 35511 Egypt; 2 Department of Medical Biochemistry, Mansoura University, Mansoura, Egypt; 3 Department of Anesthesia, Mansoura University, Mansoura, Egypt

**Retraction Note: BMC Pregnancy Childbirth 17, 160 (2017)**

**https://doi.org/10.1186/s12884-017-1349-x**

The Editor has retracted this article as the authors were unable to provide evidence that they have received ethics approval for the full duration of this study in the hospital setting and were unable to provide evidence that they have received ethics approval for the parts of the study done in private settings. Abd Elaziz A. El Refaeey and Abdelhady Zaied do not agree to this retraction. Hosam Abdelfattah, Alaa Mosbah, Anas M. Gamal, Emad Fayla, Waleed Refaie, Rafik I. Barakat, Amal K. Seleem and Mohammed Maher have not responded to any correspondence from the editor/publisher about this retraction.

